# Nonlinear relationship between blood urea nitrogen to albumin ratio and mortality risk in older patients with cerebrovascular and cardiovascular diseases: An NHANES analysis

**DOI:** 10.1371/journal.pone.0334538

**Published:** 2025-10-27

**Authors:** Li Li, Chengbo Li, Jiang Zhu

**Affiliations:** Department of Neurology, Affiliated Hospital of Chengde Medical College, Cheng de, China; Shaanxi Provincial People's Hospital, CHINA

## Abstract

**Background:**

The blood urea nitrogen to albumin ratio (BAR) has emerged as a potential prognostic biomarker in elderly patients with cardiovascular and cerebrovascular diseases (CVDs). This study investigates the association between BAR and all-cause as well as cardiac mortality in this population.

**Methods:**

We analyzed data from 4,113 elderly CVDs patients derived from the National Health and Nutrition Examination Survey (NHANES), with a mean follow-up of 82.4 months. Participants were categorized into three BAR groups: T1 (<3.55), T2 (3.55–5.00), and T3 (≥5.00). Weighted multivariable Cox regression assessed the association between BAR and all-cause mortality. The Fine and Gray competing risks model evaluated cardiac mortality, accounting for competing events. Hazard ratios (HRs) were calculated for continuous and categorical BAR. Subgroup, threshold effect, and sensitivity analyses were performed to confirm the robustness and explore nonlinear relationships.

**Results:**

During follow-up, 2,178 all-cause and 752 cardiac deaths occurred. Continuous BAR was significantly associated with increased all-cause mortality (HR = 1.10, 95% CI: 1.07–1.13, p < 0.001). Compared to T1, the highest BAR group (T3) showed elevated all-cause mortality risk (HR = 1.33, 95% CI: 1.16–1.53, p < 0.001). Each unit increase in BAR corresponded to a 9% increase in all-cause mortality and a 14% increase in cardiac mortality. Threshold analysis revealed a nonlinear association with increased risk above specific BAR levels. Subgroup and sensitivity analyses further validated these findings.

**Conclusion:**

BAR is a significant and independent predictor of all-cause and cardiac mortality in elderly patients with CVDs. Incorporation of BAR into clinical risk assessment may help improve identification of high-risk patients and support targeted interventions.

## Introduction

Cerebrovascular and cardiovascular diseases (CVDs) remain critical health challenges in the global aging population [1]. The increasing complexity of clinical management in these conditions necessitates the development of robust prognostic indicators to optimize treatment strategies and patient outcomes [2].

Blood urea nitrogen (BUN) is a marker of renal function and protein metabolism [3], with elevated levels linked to adverse outcomes in heart failure, stroke, and other conditions [4–6]. Serum albumin reflects nutritional and inflammatory status, and low levels are associated with poor prognosis and increased mortality [7–11]. The blood urea nitrogen to serum albumin ratio (BAR) integrates these parameters and may offer a more comprehensive assessment of patient health [12–14].

While BUN and albumin individually have established prognostic value, the utility of BAR in elderly patients with cerebrovascular and cardiovascular diseases remains underexplored. Recent studies suggest BAR predicts mortality in critical care and chronic illness settings [15–18], but its role in this demographic requires further investigation.

Our study aims to investigate the association between BAR and mortality in elderly cardiovascular patients using NHANES data, seeking to enhance early risk identification and clinical intervention strategies.

## Methods

### Study population

The National Health and Nutrition Examination Survey (NHANES) is a nationwide survey conducted by the Centers for Disease Control and Prevention(CDC), aimed at assessing the health and nutritional status of residents of the United States. It collects data through interviews and physical examinations, offering insights into chronic diseases and health risks that are vital for public health research. The NHANES protocols were reviewed and approved by the Research Ethics Review Board of the National Center for Health Statistics, and written informed consent was obtained from all participants prior to their involvement in the survey.

**Fig 1:**
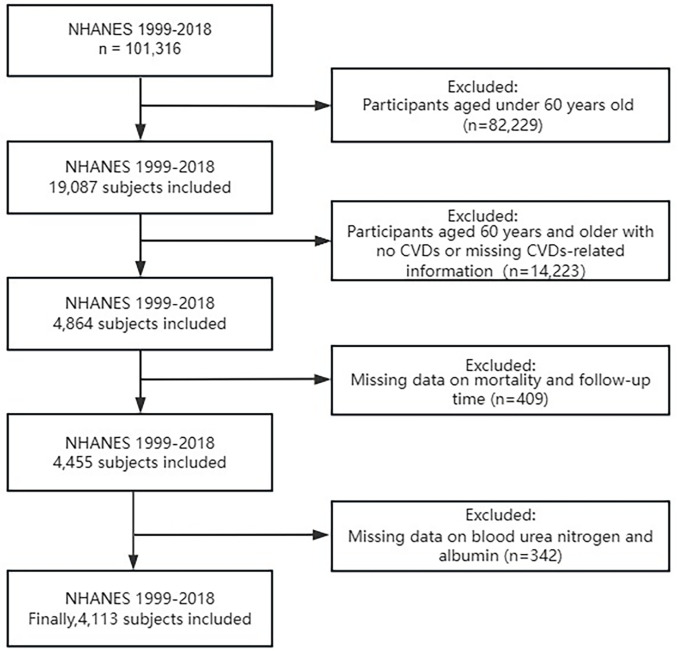
Flow diagram of participant selection in the National Health and Nutrition Examination Survey (NHANES) 1999-2018.

This study employed data from the NHANES for the period 1999–2018, initially comprising 101,316 participants. The selection process is outlined in Fig 1. Participants under 60 years old were excluded (n = 82,229). Then, among participants aged 60 years or older, those with no CVDs or missing CVD-related information were further excluded (n = 14,223), resulting in 4,864 eligible subjects.Subsequently, 409 participants with incomplete mortality and follow-up data were excluded, leaving 4,455 subjects for further analysis. Finally, 342 individuals were excluded due to missing blood urea nitrogen (BUN) and albumin data. Ultimately, 4,113 eligible subjects were included for further analysis.

Participants were systematically excluded based on predefined criteria: (a) age < 60 years, (b) absence of cardiovascular and cerebrovascular diseases (CVDs), (c) missing mortality and follow-up data, and (d) incomplete blood urea nitrogen (BUN) and albumin measurements.

### Definition of cerebrovascular and cardiovascular diseases

An individual was classified as having cerebrovascular and cardiovascular diseases if they answered “yes” to the question: “Has a medical practitioner ever diagnosed you with coronary heart disease/angina/heart attack, congestive heart failure, or stroke?” This definition is consistent with that used in previous studies, including reference [19], which employed the same standard question to ensure comparability.

### Outcome definition

Mortality data were obtained by linking NHANES participants’ unique identifiers with the National Death Index (NDI), which provides exact dates of death when available. This linkage allows precise calculation of survival time from the date of baseline examination to the date of death or censoring. For participants enrolled in later years, specifically those in 2018, follow-up was truncated at December 31, 2019, resulting in a maximum follow-up duration of approximately one year for this subgroup. The choice of follow-up cutoff was primarily driven by data availability and the need to balance maximal follow-up length with the completeness and quality of mortality ascertainment. While following all participants over a fixed period or until average life expectancy may reduce bias, such approaches are limited by data structure and cohort entry times in NHANES. We discuss these limitations and their potential impact on our findings in the Conclusion section.Cardiovascular(CAD) Mortality was defined according to the International Classification of Diseases, Tenth Revision (ICD-10) codes specifically related to heart disease: I00-I09 (rheumatic heart diseases), I11 (hypertensive heart disease), I13 (hypertensive heart and kidney disease), and I20-I51 (ischemic heart diseases and other forms of heart disease).

### Covariates

The covariates include sociodemographic information, examination and testing data, vascular disease risk factors. Sociodemographic information was collected through questionnaires, primarily including age, sex, race, educational level, the family income to poverty ratio (PIR), and marital status. The PIR is defined as the ratio of family income to the poverty threshold specific to family size, year, and state. Participants were divided into two categories based on their income: low income (PIR < 1.13) and high income (PIR ≥ 1.13). Marital status was categorised as follows: “married/living with a partner” or “widowed/divorced/separated/never married”. Examination and testing data encompass body mass index (BMI), and creatinine. Body Mass Index (BMI) Classification: Participants were categorised according to the World Health Organization (WHO) standards: normal weight (≤24.9 kg/m^2^), overweight (25–29.9 kg/m^2^), and obese (≥30 kg/m^2^). Creatinine levels were obtained through blood tests.Vascular disease risk factors include hypertension, diabetes, hyperlipidemia, smoking, and alcohol consumption. Hypertension (HT) is defined as follows: 1. Self-reported diagnosis of hypertension as informed by healthcare professionals or taking medications to lower blood pressure; 2. The average blood pressure data meets the standards of systolic blood pressure not falling below 140 mmHg and/or diastolic blood pressure not less than 90 mmHg. Diabetes is defined by the following criteria: 1. Self-reported diagnosis by medical professionals or history of antidiabetic drug use; 2. Biochemical indicators meeting any of the following standards: fasting blood glucose level of 126 mg/dL or above; HbA1c equal to 6.5% or higher; the Oral Glucose Tolerance Test(OGTT) test shows that the 2-hour blood glucose is not less than 200 mg/dL. Likewise, if a participant meets any of the following criteria, they are considered to have hyperlipidemia: self-reported doctor-informed diagnoses or use of lipid-lowering medications; fasting triglyceride levels equal to 150 mg/dL or higher; HDL cholesterol less than 40 mg/dL. Such standards are defined based on recommendations from the National Cholesterol Education Program. Smoking Classification: Participants who had smoked at least 100 cigarettes in their lifetime were classified based on current smoking status. Those answering “every day” or “some days” to “Do you/Does the sample person now smoke cigarettes?” were defined as current smokers; those answering “never” were former smokers. Participants who smoked less than 100 cigarettes lifetime were classified as non-smokers. This classification approach is consistent with that used in multiple previously published studies [20] based on the NHANES database.Alcohol Consumption Classification: Participants were stratified into three groups based on self-reported drinking status: Never Drinkers, Moderate Drinkers, and Heavy Drinkers. Never Drinkers were defined as individuals who have not consumed a minimum of 12 servings of any alcoholic beverage in their lifetime. Moderate Drinkers were defined as individuals who have consumed one alcoholic beverage per day for women or a maximum of two drinks per day for men over the past year. Heavy Drinkers were defined as women who exceed one drink daily, men who exceed two drinks daily, or those who have ever consumed more than four alcoholic beverages in a single day. These definitions are consistent with the standards from the National Institute on Alcohol Abuse and Alcoholism (NIAAA) and reference the work of Zhi-Qin Xie et al. [21]. For further details, please visit the NIAAA official website: http://www.niaaa.nih.gov/alcohol-health/overview-alcohol-consumption/moderate-binge-drinking.

### Calculation of BAR

The formula for BAR is as follows:BAR(mg/g)= BUN (mg/dl)/ALB (g/dl). In the course of our data analysis, we not only evaluated BAR as a continuous variable, but also categorised participants into three groups based on the tertile distribution of BAR values: T1 (BAR < 3.55), T2 (3.55–5.00), and T3 (BAR ≥ 5.00). This stratification approach ensured relatively balanced sample sizes across groups (T1: 33.09%; T2: 32.31%; T3: 34.60%), thereby enhancing the reliability of the statistical analyses and the robustness of the findings. Furthermore, the grouping criteria were informed by previous research [13], which has demonstrated that mortality risk significantly increases when BAR values exceed certain thresholds, providing clinical reference for the classification.

### Statistical analysis

Continuous variables were presented as mean ± standard deviation, while categorical variables were reported as frequency counts with weighted percentages (%). Missing data was explicitly documented for each variable. The Wilcoxon rank-sum test was used to compare continuous variables between groups, and the Chi-square test was employed for categorical variables. The BAR was analyzed both as a categorical variable (divided into tertiles) and as a continuous variable. The relationship between BAR and all-cause mortality among patients with cardiovascular diseases was investigated using weighted multivariable Cox regression. The association between BAR and cardiac-related mortality in this population was explored using the Fine and Gray competing risks model, with deaths from other causes treated as competing events. The statistical analyses were conducted using the R language (version 4.4.1) and the EasyFit software (version 4.2). Curve fitting and threshold effect analyses were conducted using the EasyFit software, version 4.2. All statistical analyses were evaluated using p-values (with a significance level set at 0.05) and confidence intervals to ensure the scientific validity and reliability of the results.

## Results

This study included 4,113 participants with a mean follow-up duration of 82.4 months, during which a total of 2,178 all-cause mortality cases were recorded, 752 of which were specifically attributed to cardiovascular diseases; a comprehensive overview of the demographic and clinical characteristics of the cohort is presented in Table 1.

**Table 1:** Baseline characteristics of three groups.

Variable	T1 (BAR < 3.55)	T2 (3.55–5.00)	T3 (≥5.00)	P-value
Sample size, n	1361	1329	1423	
Age, years	70.44 ± 6.98	72.99 ± 7.24	75.22 ± 6.82	<0.001
CR,mg/dl	0.89 ± 0.21	1.03 ± 0.32	1.58 ± 1.12	<0.001
UA,mg/dl	5.53 ± 1.32	5.95 ± 1.43	6.78 ± 1.87	<0.001
MeanFU,months	97.61 ± 58.54	85.83 ± 56.89	64.66 ± 49.52	<0.001
Gender, male	765 (51%)	817 (58%)	833 (53%)	0.012
Race				0.008
Mexican American	193 (3.6%)	148 (2.8%)	124 (2.8%)	
Other Hispanic	87 (3.2%)	83 (2.6%)	64 (2.0%)	
Non-Hispanic White	712 (77%)	814 (81%)	929 (83%)	
Non-Hispanic Black	289 (9.7%)	204 (6.8%)	243 (8.6%)	
Other Race	80 (6.4%)	80 (6.8%)	63 (3.9%)	
Education				0.7
<High school	276 (12%)	282 (12%)	253 (12%)	
Completed High school	263 (16%)	205 (13%)	246 (15%)	
>High school	817 (72%)	838 (75%)	919 (72%)	
Missing for Education	5 (0.1%)	4 (0.2%)	5 (0.2%)	
Marital,Married/Living with partner	756 (60%)	766 (63%)	726 (54%)	<0.001
Missing for Marital	17 (1.0%)	10 (0.5%)	6 (0.3%)	
BMI_group				0.009
normal	328 (23%)	287 (21%)	317 (22%)	
overweight	495 (36%)	489 (35%)	442 (31%)	
obesity	491 (38%)	501 (40%)	558 (40%)	
Missing for BMI_group	47 (3.6%)	52 (2.9%)	106 (6.5%)	
PIR_group, low	374 (19%)	289 (14%)	291 (15%)	0.022
Missing for PIR_group	116 (8.4%)	114 (7.6%)	138 (8.6%)	
Hypertension, yes	1073 (77%)	1040 (76%)	1211 (85%)	<0.001
Missing for Hypertension	0 (0%)	1 (<0.1%)	0 (0%)	
Diabetes,yes	482 (31%)	514 (37%)	696 (46%)	<0.001
Missing for Diabetes	36 (2.5%)	17 (1.0%)	27 (2.3%)	
Hyperlipidemia,yes	1044 (79%)	1006 (79%)	1083 (77%)	0.8
Missing for Hyperlipidemia	55 (2.4%)	53 (2.9%)	54 (3.2%)	
Smoking				<0.001
non-smokers	527 (39%)	538 (41%)	596 (42%)	
former smokers	549 (42%)	632 (46%)	707 (50%)	
current smokers	283 (19%)	158 (13%)	120 (8.1%)	
Missing for Smoking	2 (<0.1%)	1 (<0.1%)	0 (0%)	
Drinking				0.001
never	204 (15%)	198 (13%)	220 (15%)	
moderate	316 (28%)	367 (32%)	367 (29%)	
heavy	432 (28%)	320 (25%)	294 (19%)	
Missing for Drinking	409 (29%)	444 (31%)	542 (36%)	
All-cause mortality,yes	608 (44%)	669 (44%)	901 (60%)	<0.001
CAD mortality,yes	198 (14%)	215 (14%)	339 (23%)	<0.001

1. MeanFU: mean follow-up time.

2. Mean ± SD for continuous; n (%) for categorical.

3.Categorical variables were analyzed by Chi-square test; continuous variables by Wilcoxon rank-sum test.

Table 1 summarizes the baseline characteristics of the 4,113 participants stratified by BAR into three groups: T1 (BAR < 3.55), T2 (3.55–5.00), and T3 (BAR ≥ 5.00). Stratified by BAR groups, the mean follow-up times were 97.6 months for T1 (BAR < 3.55), 85.8 months for T2 (BAR 3.55–5.00), and 75.2 months for T3 (BAR ≥ 5.00), with statistically significant differences observed among the groups (P < 0.001). Differences in follow-up duration among BAR categories were observed and should be taken into account when interpreting mortality outcomes. Significant differences in age were found across the groups (P < 0.001), as well as in creatinine and uric acid levels (both P < 0.001).Males accounted for 54% of participants, with variations among groups (P = 0.012). Most participants were Non-Hispanic White (80%), with differences in racial composition (P = 0.008). Educational attainment was similar across groups (P = 0.7).Marital status and economic level (PIR) both showed significant differences among groups (approximately 40% widowed or divorced, and higher prevalence of low PIR < 1.13 in T1, with P < 0.001 and P = 0.022, respectively). Considering their potential impact on survival outcomes, these factors were included as covariates in all multivariate analyses to control for confounding effects.Body mass index categories revealed a higher prevalence of obesity in T3 (P = 0.009). Hypertension (79%, P < 0.001) and diabetes (38%, P < 0.001) were common. Hyperlipidemia prevalence was high and consistent across groups (P = 0.8). Smoking status and alcohol consumption differed significantly among groups (P < 0.001 and P = 0.001, respectively). The overall all-cause mortality rate was 49%, with significant group differences (P < 0.001); cardiovascular mortality affected 17% (P < 0.001).These findings comprehensively describe the cohort’s baseline characteristics and underpin the adjusted analyses assessing the relationship between BAR and mortality.

Table 2 presents the association between BAR and all-cause mortality among elderly patients with cardiovascular and cerebrovascular diseases, focusing on Model 3, which adjusts for demographic and clinical covariates. The continuous BAR is significantly associated with all-cause mortality, showing a hazard ratio (HR) of 1.10 (95% CI: 1.07–1.13, p < 0.001). Categorized by BAR tertiles, T3 (BAR ≥ 5.00) exhibits a significantly higher mortality risk (HR = 1.33; 95% CI: 1.16–1.53) relative to the reference group T1 (BAR < 3.55), while T2 shows no significant difference (HR = 1.00; 95% CI: 0.89–1.13). A significant trend across tertiles is confirmed (p for trend < 0.001), indicating a dose-response relationship between BAR and mortality risk.

**Table 2:** Association between BAR and All-cause mortality.

BAR (mg/g)	HR (95%CI) P-value		
	Model 1	Model 2	Model 3
Continuous	1.11(1.09–1.14) < 0.001	1.14 (1.11–1.16) < 0.001	1.10(1.07–1.13) < 0.001
T1	Ref	Ref	Ref
T2	1.18(1.04-1.33)	1.05(0.92-1.19))	1.00(0.89-1.13))
T3	2.22 (1.96-2.53)	1.63(1.43-1.86)	1.33(1.16-1.53)
p for trend	<0.001	<0.001	<0.001

Model 1, Unadjusted; Model 2, adjusted for Age, Gender, Race, Education, Marital, PIR_group; Model 3, adjusted for Age, Gender, Race, Education, Marital, PIR_group, BMI_group, Hypertension, Diabetes, Hyperlipidemia, smoking, drinking, CR and UA.

Further analysis in Table 3 explores the linear and threshold effects of BAR on all-cause and cardiac mortality. Model I confirms that each unit increase in BAR is associated with increased risks—9% for all-cause mortality (HR = 1.09, 95% CI: 1.07–1.12, p < 0.0001) and 14% for cardiac mortality (HR = 1.14, 95% CI: 1.10–1.18, p < 0.0001). Model II introduces threshold analyses, revealing that below BAR thresholds of 2.69 (all-cause) and 2.27 (cardiac), the hazard ratios suggest a possible protective effect, though only the all-cause association reaches statistical significance (p = 0.0479). Above these thresholds, risks significantly increase—HRs of 1.10 (all-cause) and 1.08 (cardiac), both with p < 0.0001. Effect difference analyses confirm that mortality risks above the thresholds are significantly elevated, corroborated by likelihood ratio tests (all-cause p = 0.007; cardiac p = 0.019).

**Table 3:** Association between BAR and mortality.

Outcome:	All-cause mortality	Cardiac mortality
Model I		
Linear effect	1.09 (1.07, 1.12) <0.0001	1.14 (1.10, 1.18) <0.0001
Model II		
Threshold (K)	2.69	2.27
Effect 1 (< K)	0.80 (0.65, 1.00) 0.0479	0.45 (0.13, 1.54) 0.202
Effect 2 (> K)	1.10 (1.08,1.12) <0.0001	1.08(1.04,1.12) <0.0001
Effect Difference (2 vs 1)	1.37 (1.10, 1.71) 0.0050	2.19 (1.21, 3.98) 0.0101
Likelihood Ratio Test	0.007	0.019

Data in the table:HR (95%Cl) P value.

Outcome variables: All-cause mortality, Cardiac mortality.

Exposure variable: BAR.

Adjustment variables: Age, Gender, Race, Education, Marital, PIR_group,BMI_group, Hypertension, Diabetes, Hyperlipidemia, smoking, drinking, CR and UA.

**Fig 2:**
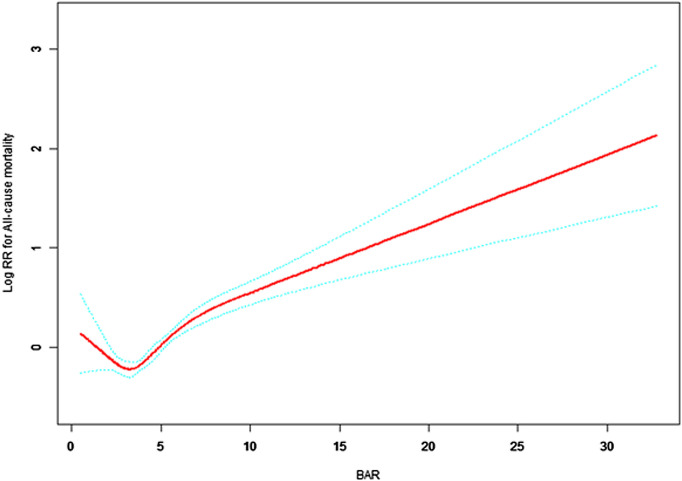
Association between BAR and log relative risk of all-cause mortality in elderly cardiovascular and cerebrovascular patients. Solid red line shows estimated log relative risk (RR); dashed blue lines indicate 95% confidence intervals. Mortality risk remains relatively low when BAR is approximately below 3, and tends to increase above this value. X-axis:BAR (blood urea nitrogen to albumin ratio). Y-axis: Log relative risk of all-cause mortality.

**Fig 3:**
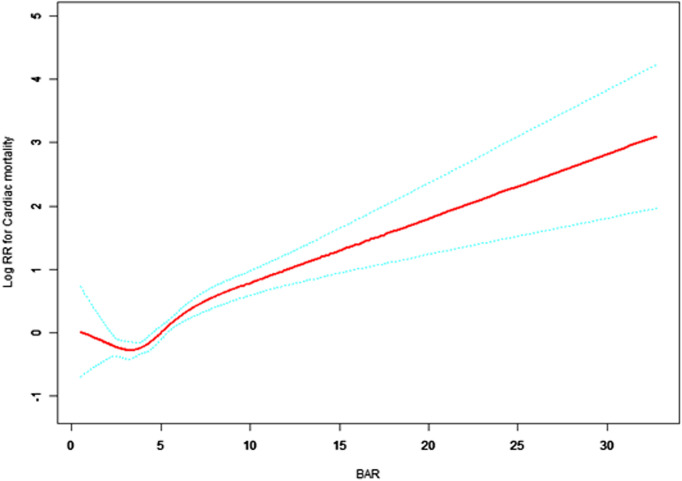
Association between BAR and log relative risk of cardiac mortality in elderly cardiovascular and cerebrovascular patients. Solid red line represents estimated log relative risk (RR); dashed blue lines show 95% confidence intervals. Cardiac mortality risk remains relatively stable when BAR is roughly below 3, with an increasing trend observed beyond this point. X-axis: BAR (blood urea nitrogen to albumin ratio). Y-axis: Log relative risk of cardiac mortality.

Figs 2 and 3 illustrate these findings, demonstrating a nonlinear relationship between BAR and mortality risks. Mortality rates remain relatively stable below a BAR of approximately 3, but rise sharply thereafter, particularly for cardiac mortality. These results underscore the utility of BAR as a prognostic biomarker for mortality in elderly patients with cardiovascular and cerebrovascular diseases, highlighting a critical threshold that may inform clinical risk stratification.

Table 4 shows the results of the subgroup analyses, demonstrating that the association between BAR and all-cause mortality remains consistent across different patient subgroups. These subgroups are stratified by relevant clinical and demographic factors, and the effect sizes align with the primary findings reported in Table 2. This further supports the robustness of the observed relationship between BAR and mortality risk in elderly patients with cardiovascular and cerebrovascular diseases.

**Table 4:** Results of subgroup analysis.

Subgroup	N	Effect Estimate (HR)	95%CI	P-value
Gender				
Male	2415	1.14	1.12–1.15	<0.0001
Female	1698	1.14	1.12–1.16	<0.0001
Age,years				
≤ 68	1304	1.14	1.12–1.17	<0.0001
> 69 and <77	1334	1.12	1.10–1.15	<0.0001
≥ 78	1475	1.14	1.11–1.17	<0.0001
Race				
Mexican American	465	1.17	1.12–1.23	<0.0001
Other Hispanic	234	1.19	1.12–1.27	<0.0001
Non-Hispanic White	2455	1.16	1.14–1.18	<0.0001
Non-Hispanic Black	736	1.11	1.09–1.13	<0.0001
Other Race	223	1.23	1.12–1.35	<0.0001
Education				
< High school	811	1.17	1.13–1.20	<0.0001
Completed High school	714	1.11	1.09–1.13	<0.0001
> High school	2574	1.16	1.14–1.18	<0.0001
Marital				
Widowed/Divorced/Separated/Never married	1832	1.13	1.11–1.15	<0.0001
Married/Living with partner	2248	1.15	1.13–1.17	<0.0001
PIR_ group				
< 1.13	954	1.14	1.11–1.17	<0.0001
>=1.13	2791	1.14	1.12–1.15	<0.0001
BMI_group				
normal	932	1.18	1.15–1.22	<0.0001
overweight	1426	1.15	1.12–1.19	<0.0001
obesity	1550	1.14	1.12–1.16	<0.0001
Hypertension				
no	788	1.26	1.21–1.32	<0.0001
yes	3324	1.13	1.12–1.15	<0.0001
Diabetes				
no	2341	1.17	1.15–1.19	<0.0001
yes	1692	1.12	1.10–1.14	<0.0001
Hyperlipidemia				
no	818	1.16	1.12–1.20	<0.0001
yes	3133	1.14	1.13–1.16	<0.0001
Smoking				
non-smokers	1661	1.16	1.14–1.19	<0.0001
former smokers	1888	1.13	1.11–1.15	<0.0001
current smokers	561	1.12	1.08–1.16	<0.0001
Drinking				
never	622	1.10	1.07–1.13	<0.0001
moderate	1050	1.22	1.18–1.26	<0.0001
heavy	1046	1.13	1.11–1.16	<0.0001

In order to evaluate the impact of missing data on the findings, a sensitivity analysis was performed by excluding participants with any missing values across all variables. This process yielded a final analytical sample of 2,255 elderly patients with cardiovascular and cerebrovascular diseases. We re-estimated the association between BAR and all-cause mortality in this specific population using this complete dataset, and the results were consistent with our primary analysis (see Supplementary Table 1 in S1 file). When BAR was analysed as a continuous variable, it remained associated with all-cause mortality (OR=1.11, 95% CI: 1.08–1.15, P < 0.0001), indicating stability in the observed relationship regardless of the data handling methods employed. Furthermore, when BAR was divided into tertiles, participants in the highest tertile (T3) exhibited a higher odds ratio of mortality compared to those in the lowest tertile (T1) (OR=1.22, 95% confidence interval: 1.02–1.45, P = 0.027). The comparison across tertiles also exhibited a trend that was consistent with increasing levels of BAR (P for trend = 0.026). All analyses were adjusted for the same set of covariates as detailed in Model 3 of Table 2, ensuring consistency within our analytical approach.

To further address potential bias related to follow-up duration, we conducted a sensitivity analysis by restricting the cohort to participants with a minimum follow-up period of one year. After excluding 210 participants from the original sample of 4,113, a total of 3,903 elderly patients with cardiovascular and cerebrovascular diseases were included in this sensitivity analysis. The results are shown in Supplementary Table 2 in S1 file. The findings were similar to those observed in Supplementary Table 1 in S1 file, with slight variations, indicating that the association between BAR and all-cause mortality remained generally robust. This additional sensitivity analysis further supports the stability and reliability of our primary findings despite differences in follow-up time.

## Discussion

Our study demonstrates the significant prognostic value of BAR in elderly patients with cardiovascular and cerebrovascular diseases. Specifically, each unit increase in BAR corresponds to a 9% rise in all-cause mortality risk and a 14% increase in cardiac mortality. Notably, a threshold effect was identified around a BAR value of 3, below which mortality risk remains comparatively low, whereas values above this demarcation indicate markedly increased risk. This threshold effect expands on prior research by Zhang et al. [22] and Dundar et al. [23], emphasizing the need for nuanced clinical interpretation of BAR values.

From a biological perspective, BAR integrates two pathophysiologically relevant parameters—renal function (reflected by BUN) and nutritional/inflammatory status (reflected by albumin). Elevated BUN levels may not only indicate impaired renal perfusion commonly seen in elderly patients with multiple comorbidities but also reflect neurohormonal activation states associated with disease severity [24–27]. Similarly, Aslan and Ardahanlı [28]highlighted uric acid as an important metabolic factor in cardiovascular risk, underscoring the complex interplay of renal and metabolic dysfunction in cardiovascular pathology. Conversely, albumin functions crucially in maintaining oncotic pressure and exhibits antioxidant properties; reductions in albumin levels (hypoalbuminemia) are linked to inflammation, malnutrition, and adverse cardiovascular outcomes [29–32]. Consistent with this, Ardahanli and Özmen [33]reported that albumin-based ratios, like the C-reactive protein to albumin ratio, serve as sensitive inflammatory markers in cardiovascular disease, reinforcing albumin’s role in reflecting systemic inflammation. Thus, BAR serves as a composite biomarker capturing multi-organ vulnerability—encompassing renal impairment and systemic inflammation—both key contributors to mortality in this high-risk population. It is evident that, given the fact that the study cohort has been directly derived from the general population as represented by the NHANES dataset, BAR shows considerable promise as a practical and cost-effective screening tool in primary care. Its simplicity, cost-effectiveness, and ability to reflect systemic physiological derangements make BAR an attractive candidate for early risk stratification, potentially guiding intensified monitoring or intervention before the occurrence of overt clinical deterioration. In summary, the present study enhances extant literature by delineating the prognostic threshold of BAR and elucidating its biological underpinnings involving renal and inflammatory pathways. It is recommended that future prospective studies further explore the mechanistic links and validate the utility of BAR across diverse populations, including community-dwelling elderly individuals, with a view to facilitating its integration into routine clinical practice.

Our study has several strengths and limitations. One of the main strengths is the use of a large, diverse cohort, which enhances the generalisability of our findings regarding the prognostic value of the Blood Urea Nitrogen to Albumin Ratio (BAR) in elderly patients with cardiovascular and cerebrovascular disease. The comprehensive data collection allows a robust analysis of the relationship between BAR and mortality outcomes. However, our study is not without limitations. First, it is a single-centre cohort study, which limits our ability to establish causality. Importantly, the observational design lacks a control group, which may limit the strength of causal inference and increase susceptibility to unmeasured confounding.In addition, although we adjusted for several confounding factors, the potential for residual confounding remains, especially given the variability in underlying health conditions among patients. Residual confounding from unmeasured variables such as diet, inflammation markers, and other lifestyle factors remains possible and could influence the observed associations between BAR and mortality. Additionally, key confounders such as general access to healthcare, quality of medical services, and frequency of follow-up were not accounted for due to data limitations, which may also impact both BAR levels and mortality outcomes. Furthermore, defining cardiovascular and cerebrovascular diseases solely by self-report may introduce misclassification bias, as it may not fully capture clinical or subclinical conditions. Moreover, hypertension and diabetes definitions partly rely on self-reported diagnosis combined with clinical measurements, which may also lead to misclassification. Specifically, for diabetes, the diagnosis based on a single biochemical measurement or self-report—consistent with widely accepted protocols using the NHANES database—may contribute to potential misclassification due to lack of confirmatory testing. This limitation could affect the accuracy of diabetes status classification and thereby influence observed associations.This potential misclassification could affect the validity of the observed associations. Such potential misclassification can reduce the precision of our estimates and should be considered when interpreting the results. Moreover, the evaluation of BAR was based solely on its initial measurement, without accounting for potential fluctuations over time. It is acknowledged that alterations in BAR during the course of the disease or treatment interventions may have a substantial impact on its prognostic value. However, this aspect remains unaddressed in the present analysis. It is recommended that subsequent studies encompass serial measurements of BAR in order to facilitate a more profound comprehension of its dynamic character and its correlation with patient outcomes. Finally, the lack of detailed information on diet and other lifestyle factors may introduce bias, as these elements may influence both BUN and albumin levels. Additionally, socioeconomic status indicators beyond PIR, psychological factors, and healthcare utilization patterns were not available, limiting our ability to fully capture the complexity of factors impacting BAR and mortality. Future research should aim to address these limitations by incorporating multicentre prospective designs and exploring the dynamic nature of BAR in relation to patient outcomes.

## Conclusion

In conclusion, our study demonstrates that higher levels of the BAR are significantly associated with increased all-cause and cardiac mortality in elderly patients with cardiovascular and cerebrovascular diseases. These findings suggest that BAR serves as a valuable biomarker for identifying individuals at heightened risk and may inform targeted interventions to improve patient outcomes. We acknowledge that varying follow-up times, due to different enrollment periods, may affect mortality estimates. Sensitivity analysis restricting participants to at least one year of follow-up showed results largely consistent with the main findings, suggesting limited impact from follow-up duration differences. Future studies with standardized follow-up or advanced survival methods could further address potential biases.

## Supporting information

S1 File Supplementary Tables 1 and 2. (DOCX)
