# Supplementary material for: Nonlinear relationship between blood urea nitrogen to albumin ratio and mortality risk in older patients with cerebrovascular and cardiovascular diseases: An NHANES analysis
Source: PLoS One. 2025 Oct 27;20(10):e0334538. doi: 10.1371/journal.pone.0334538 (PMC12558514; doi:10.1371/journal.pone.0334538)
Supplement: S1 File — (DOCX) [file pone.0334538.s001.docx]

**Supplementary Table 1.** Relationship between BAR and All-cause mortality

| BAR (mg/g) | OR (95%CI) P-value |
| --- | --- |
| Continuous | 1.11(1.08– 1.15) <0.0001 |
| T1 | Ref |
| T2 | 1.06(0.88-1.29) 0.52 |
| T3 | 1.22(1.02-1.45) 0.027 |
| p for trend | 0.026 |

Model 4, adjusted for Age,Gender,Race,Education,Martial, PIR_group,BMI_group,Hypertension,Diabetes, Hyperlipidemia ,smoking, drinking, CR and UA

**Supplementary Table 2.** Association between BAR and All-cause mortality

| BAR (mg/g) | OR (95%CI) P-value |
| --- | --- |
| Continuous | 1.09(1.05– 1.12) <0.0001 |
| T1 | Ref |
| T2 | 1.02(0.91-1.14) 0.77 |
| T3 | 1.30(1.12-1.50) 0.0005 |
| p for trend | 0.0006 |

Model 5, adjusted for Age,Gender,Race,Education,Martial, PIR_group,BMI_group,Hypertension,Diabetes, Hyperlipidemia ,smoking, drinking, CR and UA
